# Mitophagy genes in ovarian cancer: a comprehensive analysis for improved immunotherapy

**DOI:** 10.1007/s12672-023-00750-y

**Published:** 2023-12-01

**Authors:** Wenting He, Jieping Chen, Yun Zhou, Ting Deng, Yanling Feng, Xiaolin Luo, Chuyao Zhang, He Huang, Jihong Liu

**Affiliations:** Department of Gynecologic Oncology, State Key Laboratory of Oncology in South China, Sun Yat-Sen University Cancer Center, Guangzhou, 510060 China

**Keywords:** Mitophagy, Ovarian cancer, Immunology, Tumor microenvironment, MRG_score

## Abstract

**Background:**

Mitophagy is a process of selectively degrading damaged mitochondria, which has been found to be related to immunity, tumorigenesis, tumor progression, and metastasis. However, the role of mitophagy-related genes (MRGs) in the tumor microenvironment (TME) of ovarian cancer (OV) remains largely unexplored.

**Methods:**

We analyzed the expression, prognosis, and genetic alterations of 29 MRGs in 480 OV samples. Unsupervised clustering was used to classify OV into two subtypes (clusters A and B) based on MRG changes. We compared the clinical features, differential expressed genes (DEGs), pathways, and immune cell infiltration between the two clusters. We constructed a mitophagy scoring system (MRG_score) based on the DEGs and validated its ability to predict overall survival of OV patients.

**Results:**

We found that patients with high MRG_scores had better survival status and increased infiltration by immune cells. Further analysis showed that these patients may be more sensitive to immune checkpoint inhibitor (ICI) treatment. Additionally, the MRG_score significantly correlated with the sensitivity of chemotherapeutic drugs and targeted inhibitors.

**Conclusion:**

Our comprehensive analysis of MRGs in the TME, clinical features, and patient prognosis revealed that the MRG_score is a potentially effective prognostic biomarker and predictor of treatment. This study provides new insights into the role of MRGs in OV and identifies patients who may benefit from ICI treatment, chemotherapy, or targeted treatment.

**Supplementary Information:**

The online version contains supplementary material available at 10.1007/s12672-023-00750-y.

## Introduction

Mitochondria serve as crucial sites for biological oxidation and energy conversion within eukaryotic cells. Upon experiencing damage, dysfunction, or aging, mitophagy, a form of selective autophagy, is triggered [1]. This process promotes self-degradation, the release of cytochrome C, and ultimately apoptosis, thereby ensuring the balance between the quantity and quality of mitochondria and the maintenance of normal cellular functions [1, 2]. However, the metabolic regulation mediated by mitophagy can also balance the activities of aerobic glycolysis and oxidative phosphorylation, promoting cancer cell survival [3]. Mitophagy can lead to either tumor progression or inhibition; in another words, the regulatory role of mitophagy in cancer is complex and could be cancer- and context- dependent. Some research showed that mitophagy could promote tumor progression through mechanisms such as facilitating metastasis of hepatocellular carcinoma [4], enhancing the drug resistance of small cell lung cancer [5], and supporting the survival and metabolic switch of drug-tolerant persister (DTP) cells [6]. However, some research showed the opposing results: inducing mitophagy was found to significantly inhibit the growth of chemoresistant ovarian cancer both in vitro and in vivo [7]. Therefore, additional research is required to fully elucidate the complex roles and underlying mechanisms of mitophagy in tumorigenesis.

Ovarian cancer is a type of cancer that affects the female reproductive system and has a high mortality rate, frequent recurrence, and poor prognosis. The development and progression of this cancer involve the process of mitophagy and the related genes responsible for it. A study has shown that the administration of pardaxin can activate mitophagy and induce apoptosis in ovarian cancer cells [8]. Another research has suggested that targeting epidermal growth factor receptor proteins can trigger mitophagy and cause cell death through the mammalian target of rapamycin complex 2 and Akt pathways [9]. Moreover, mutations in the ubiquitin-associated domain of p62 have been found to alter the sensitivity of ovarian cancer cells to cisplatin by promoting mitophagy through the localization of hexokinase 2 in mitochondria [10]. Nonetheless, the exact contribution of MRGs to OV remains ambiguous.

Ovarian cancer, like other solid tumors, is influenced by the tumor microenvironment (TME), which plays a crucial role in the onset, progression, spread, and recurrence of the disease [11]. While ovarian cancer is known to be an immunogenic cancer, its highly immunosuppressive TME has made immunotherapy, including immune checkpoint blockade therapy, a significant challenge [12, 13]. Studies have indicated that the success rate of immunotherapy for ovarian cancer is lower than that for other solid tumors, and the response rate of a single drug is only around 10% [14–17]. It is therefore important to understand the characteristics of the TME in ovarian cancer and identify ideal predictors of effective immunotherapy to enhance the selection of patients who may benefit from treatment. Immune infiltration refers to the phenomenon in which immune cells aggregate in the TME. Tumor-infiltrating immune cells (TIICs) are an indispensable component of the TME and have been used to predict the prognosis of cancer patients [18]. The significant infiltration of TIICs tends to coincide with favorable prognostic results in research on ovarian cancer transformation. The clinical outcomes of TIICs impact on various solid tumors were also reviewed, and it was discovered that intense infiltration of TIICs is linked to favorable clinical outcomes in a variety of cancers, including melanoma as well as head and neck, breast, bladder, urethral, ovarian, colorectal, renal, prostate, and lung cancers [19]. Increasing evidence suggests that immune therapy for cancer can enhance efficacy by inducing immune infiltration in tumors, and research into immune infiltration has become an important field in cancer immunotherapy (T cell exclusion, immune privilege, and the tumor microenvironment; The immune score as a new possible approach for the classification of cancer; Neoantigens in cancer immunotherapy) [20–22]. Studies have shown that mitophagy can impact immune infiltration and peritoneal metastasis in ovarian and endometrial cancer; however, these studies only evaluated single genes, or a single type of immune cell associated with MRGs [23, 24]. A comprehensive analysis of the characteristics of MRG-mediated TME infiltration can provide valuable insights into the underlying mechanisms of ovarian cancer development and predict the response to immunotherapy.

In this study, we conducted a comprehensive evaluation of the expression, genetic alterations, and prognosis of mitophagy-related genes (MRGs) in ovarian cancer (OV). Based on the expression of MRGs, we performed unsupervised clustering and identified two subtypes of OV, namely cluster A and B. Interestingly, the overall survival (OS) of patients in the two clusters was significantly different, suggesting the involvement of a potential mitophagy-related mechanism. To further investigate the differences between the two clusters, we analyzed their clinical features, differential expressed genes (DEGs), pathways, and immune cell infiltration. Additionally, we developed a mitophagy scoring system (MRG_score) that could predict the OS of OV patients. We also explored the relationship between MRG_score, tumor microenvironment (TME), and the efficacy of immunotherapy, chemotherapy, and targeted therapy.

## Materials and methods

### Data collection

The mRNA expression profiles and clinical data of 379 patients with ovarian cancer (TCGA-OV) were obtained from the UCSC Xena database (https://xenabrowser.net/datapages/). In addition, the dataset of 101 ovarian cancer patients (GSE63885; https://www.ncbi.nlm.nih.gov/geo/query/acc.cgi?acc=GSE63885) [25, 26] were obtained from the Gene Expression Omnibus (GEO) database (https://www.ncbi.nlm.nih.gov/geo/) [27].

The inclusion criteria of selecting GEO datasets included: (1) The experimental type of the dataset was expression profiling by array; (2) The organism of samples should be Homo sapiens; (3) The surgical samples should be derived the ovarian cancer patients diagnosed by histopathology; (4) The dataset includes a sufficient number of samples to ensure statistical power and data robustness; (5) The dataset includes complete clinical information for each sample, for example, TNM stage; tumor grade (well differentiated; moderately differentiated; poorly differentiated; undifferentiated); histopathological subtype; survival time especially overall survival; follow-up time; clinical status post chemotherapy (complete response; partial response; stable disease; progression); and clinical status at last follow-up (alive with disease; dead from disease; no evidence of disease).

Then the R packages "limma"[28] and "sva" [29] were used to correct for batch effects after merging the two datasets. Supplementary Table 1 provides detailed information on the 480 ovarian cancer patients, including clinical variables such as tumor grade, stage, follow-up time, survival status, and histopathological type.

A gene set of 29 mitophagy-related genes was obtained from the Msigdb database (http://www.gsea-msigdb.org/gsea/index.jsp), including genes such as ATG5, FUNDC1, CSNK2A2, TOMM22, CSNK2A1, MAP1LC3A, MFN2, TOMM40, MAP1LC3B, RPS27A, ATG12, UBC, TOMM70, MTERF3, PINK1, SQSTM1, UBB, MFN1, TOMM20, TOMM5, ULK1, PRKN, TOMM7, SRC, CSNK2B, VDAC1, TOMM6, UBA52, and PGAM5. A flow chart of our research work is provided in Supplementary Figure 1.

### Online analysis and software

The interaction pairs of MRGs were obtained from the STRING database (https://cn.string-db.org/) [30]. And Cytoscape (version 3.9.1) [31] was utilized to construct the protein–protein interaction (PPI) network. And the genetic alterations of MRGs, including copy number variants (CNAs) and mutations, was investigated by using the Gene Set Cancer Analysis (GSCA) database (http://bioinfo.life.hust.edu.cn/GSCA/#/) [32]. Furthermore, to predict the efficacy of immune checkpoint inhibitor (ICI) treatment the immunophenoscore (IPS) scores of ovarian cancer patients was obtained from the TCIA database (https://tcia.at/home).

### Unsupervised consensus clustering and enrichment analysis

The OV samples were consistently classified through unsupervised clustering using R package “ConsensuClusterPlus” [33]. The Gene Set Variation Analysis (GSVA) pathways, comprising HALLMARK and Kyoto Encyclopedia of Genes and Genomes (KEGG) pathways, were obtained from the Msigdb database. The pathway score for each sample was calculated using the R package "GSVA"[34] based on TCGA-OV and GSE63885 datasets. The R package "clusterprofiler"[35] was used to perform Gene Ontology (GO) and KEGG analysis of differentially expressed genes (DEGs).

### Assessment of immune cell infiltration

The ssGSEA function of R package “GSVA” [34] was used to assess the infiltration level of 23 immune cells. The marker genes of each immune cells were supplied in Supplementary Table 2. The immune cell level was further compared in variant groups.

### Construction of MRGs_score

303 differentially expressed genes (DEGs) were identified between two clusters using the R package "limma" [28] based on the criteria of |logFC|> 0.3 and P < 0.05. Among these, 51 DEGs were significantly associated with overall survival based on further univariate regression analysis (p < 0.05). To conduct and visualize principal component analysis (PCA) and generate a score for each patient, we selected the final 51 DEGs and extracted principal components 1 and 2 using R package “limma” and R package “ggplot2”. This score, termed the MRG_Score, was calculated using the following formula [36]:

$$MRG\_score\, = \,\sum\nolimits_{{}}^{{}} {\left( {PC1i} \right)} \, + \,\sum\nolimits_{{}}^{{}} {\left( {PC2i} \right)}$$

### Drug sensitivity analysis

Based on Cancer drug sensitivity Genomics (GDSC) database (www.cancerRxgene.org), the largest public resource for cancer cell drug sensitivity and drug response molecular marker information, the R package "pRRophetic" [37] was used to analyze the sensitivity of ovarian cancer patients to different chemotherapy and targeted therapy related drugs.

### Statistical analyses

All statistical analyses were implemented by R version 4.1.1. And statistical significance was set at p < 0.05 (*p < 0.05, **p < 0.01, **p < 0.001).

## Results

### Genetic alteration of MRGs

**Fig. 1:**
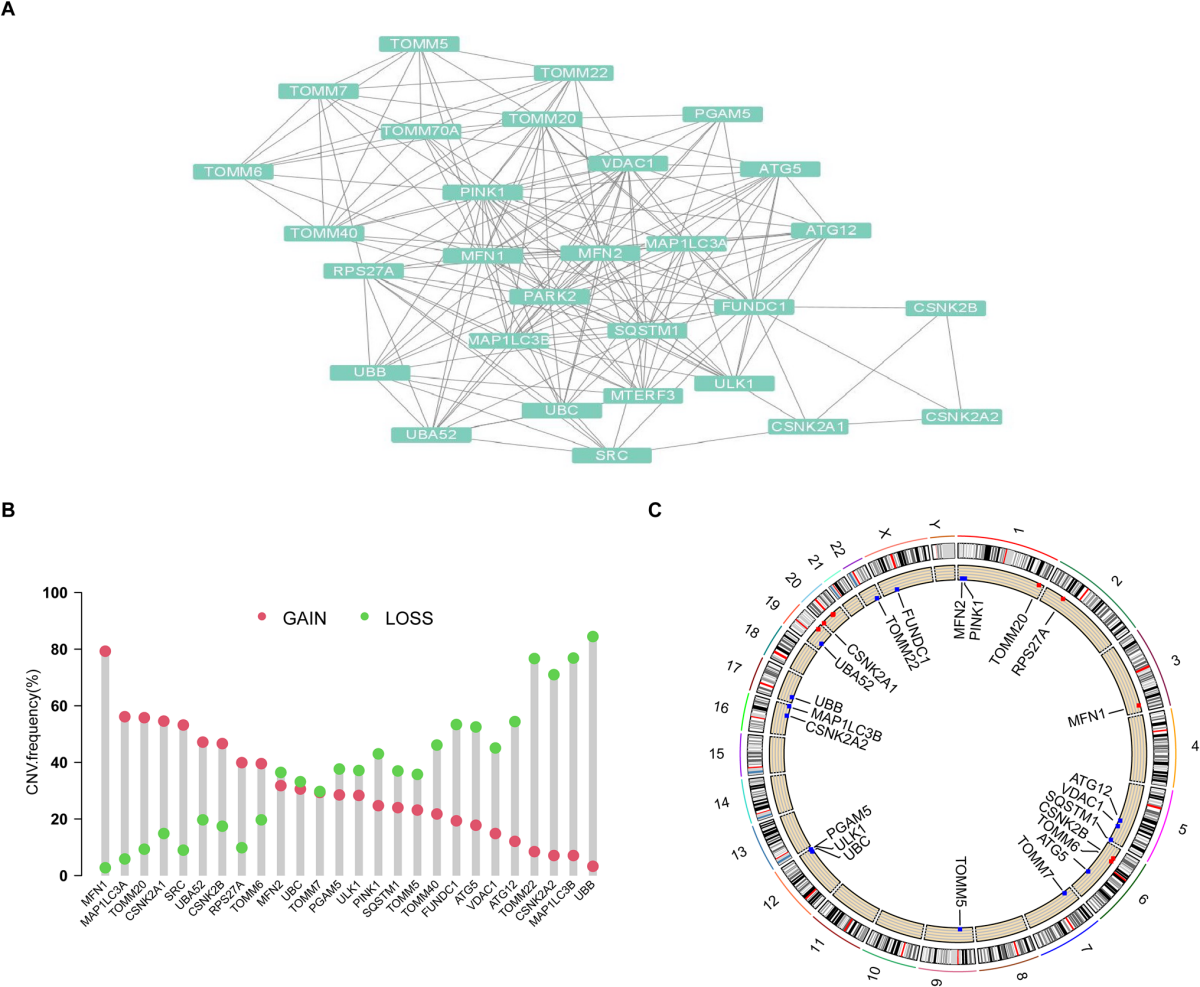
The PPI and CNA analysis of MRGs. **A** The PPI network of MRGs. **B** Frequencies of CNV gain and loss among MRGs in OV. **C** Chromosome locations of CNV alterations in MRGs

**Fig. 2:**
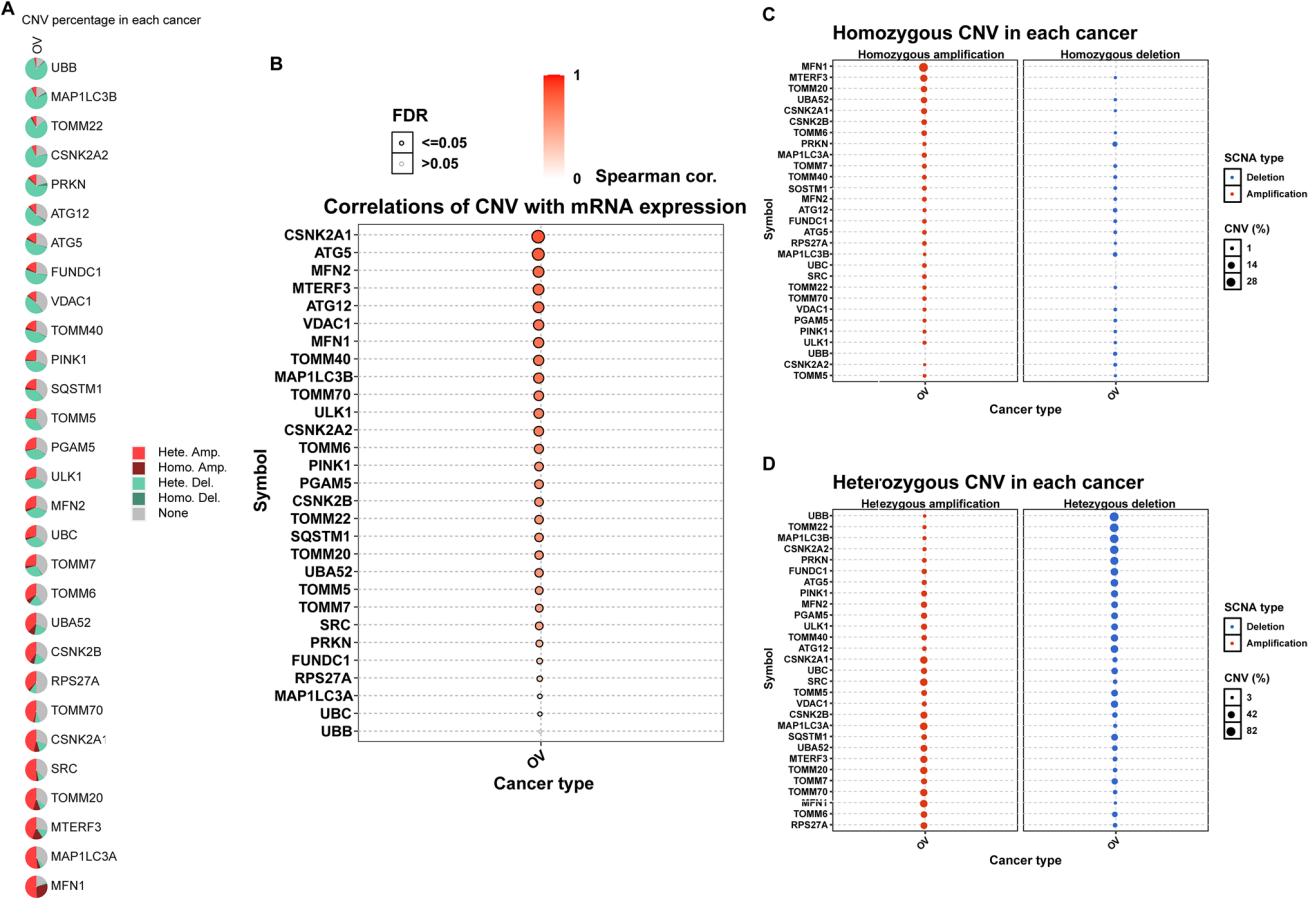
Genetic alteration of MRGs. **A** The CNA percentage of MRGs in OV. **B** The correlation of CNV with mRNA expression in OV. **C** The homozygous CNV of MRGs in OV. **D** The heterozygous CNV of MRGs in OV

In this study, 29 MRGs were curated and their potential role in ovarian cancer was investigated. Analysis of the protein–protein interaction network revealed connections between the 29 MRGs (Fig. 1A). Copy number variation (CNV) is a well-known genomic abnormality in cancer, with tumor suppressor gene inactivation or oncogene activation commonly attributed to CN loss or gain, respectively [38, 39]. Therefore, we further explored the CNV of the 29 MRGs and found that 26 MRGs had CN changes, while the remaining 3 did not have CNV information. Notably, MFN1, MAP1LC3A, TOMM20, CSNK2A1, and SRC displayed significant CNV increases, while UBB, MAP1LC3B, CSNK2A2, and TOMM22 showed CNV decreases (Fig. 1B). The chromosome localization of CNV changes in the MRGs is presented in Fig. 1C. Using the GSCA database, we found that MFN1 had the highest frequency of CNV amplification, with heterozygous amplification occurring most frequently (Fig. 2A). We also observed a positive correlation between CNV changes and mRNA expression of MRGs, with CSNK2A1 exhibiting the highest correlation (Fig. 2B). These findings suggest that MRGs may play a crucial role in tumor development in ovarian cancer.

**Fig. 3:**
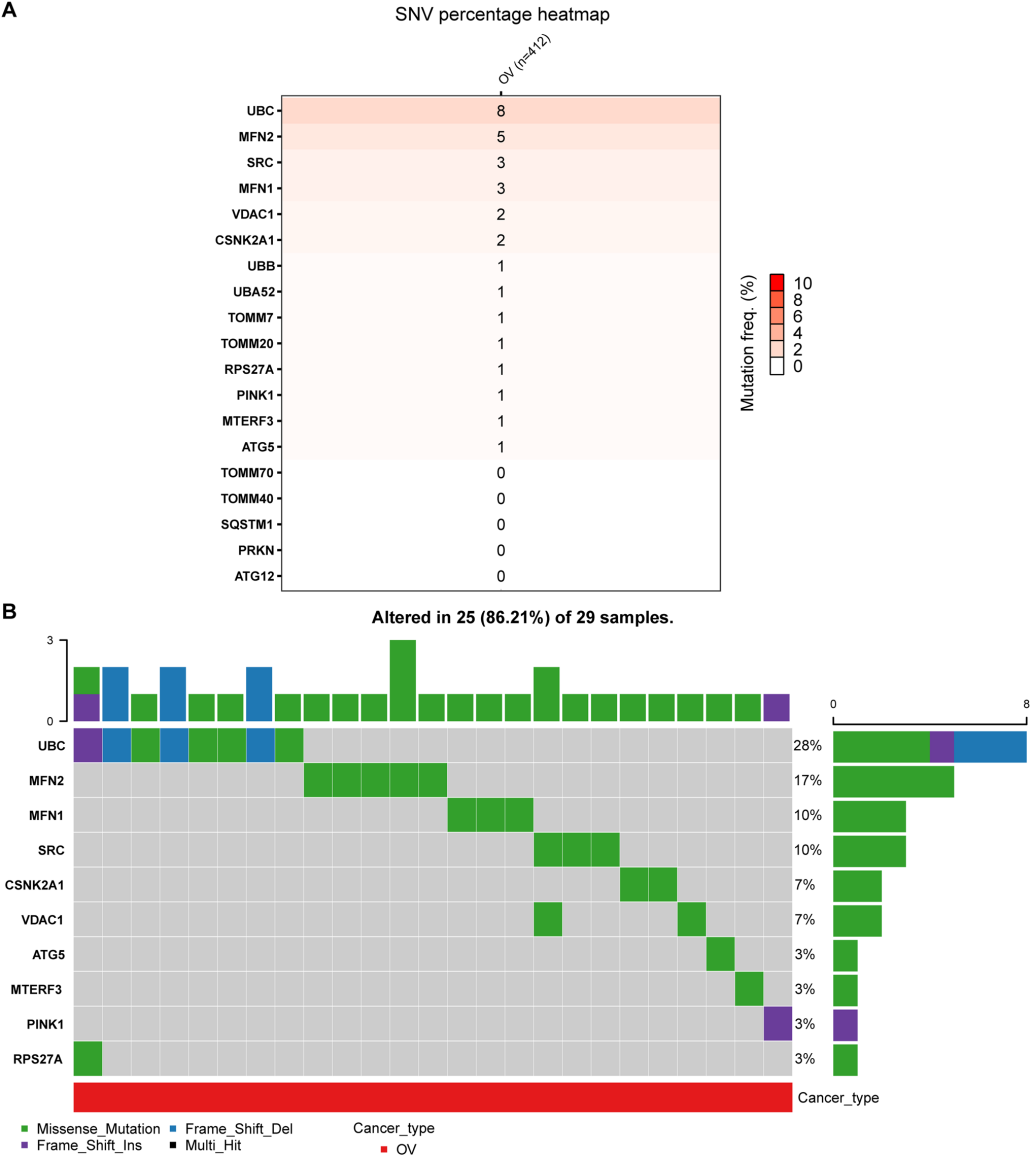
The SNV of MRGs. **A** The frequency of deleterious mutations of MRGs in OV. **B** Oncoplot presents the mutation distribution of top 10 mutated MRGs in OV

To further investigate the role of MRGs in ovarian cancer, we analyzed the percentage of homozygous and heterozygous CNVs of each MRG gene, including homozygous amplification, homozygous deletion, heterozygous amplification, and heterozygous deletion (Fig. 2C, D). In addition to CNV analysis, we explored the role of single nucleotide variants (SNVs) of MRGs in ovarian cancer. We found that UBC had the highest SNV frequency (Fig. 3A). Furthermore, we examined the distribution of mutations and classification of SNV types of the 10 most mutated MRGs in ovarian cancer (Fig. 3B). SNVs can potentially influence mRNA expression, protein stability, localization, and function, and thus assign causality [40]. Our findings suggest that MRGs may contribute to the development of ovarian cancer through both CNV and SNV mechanisms.

### Identification of mitophagy subtypes mediated by 29 MRGs in OV

**Fig. 4:**
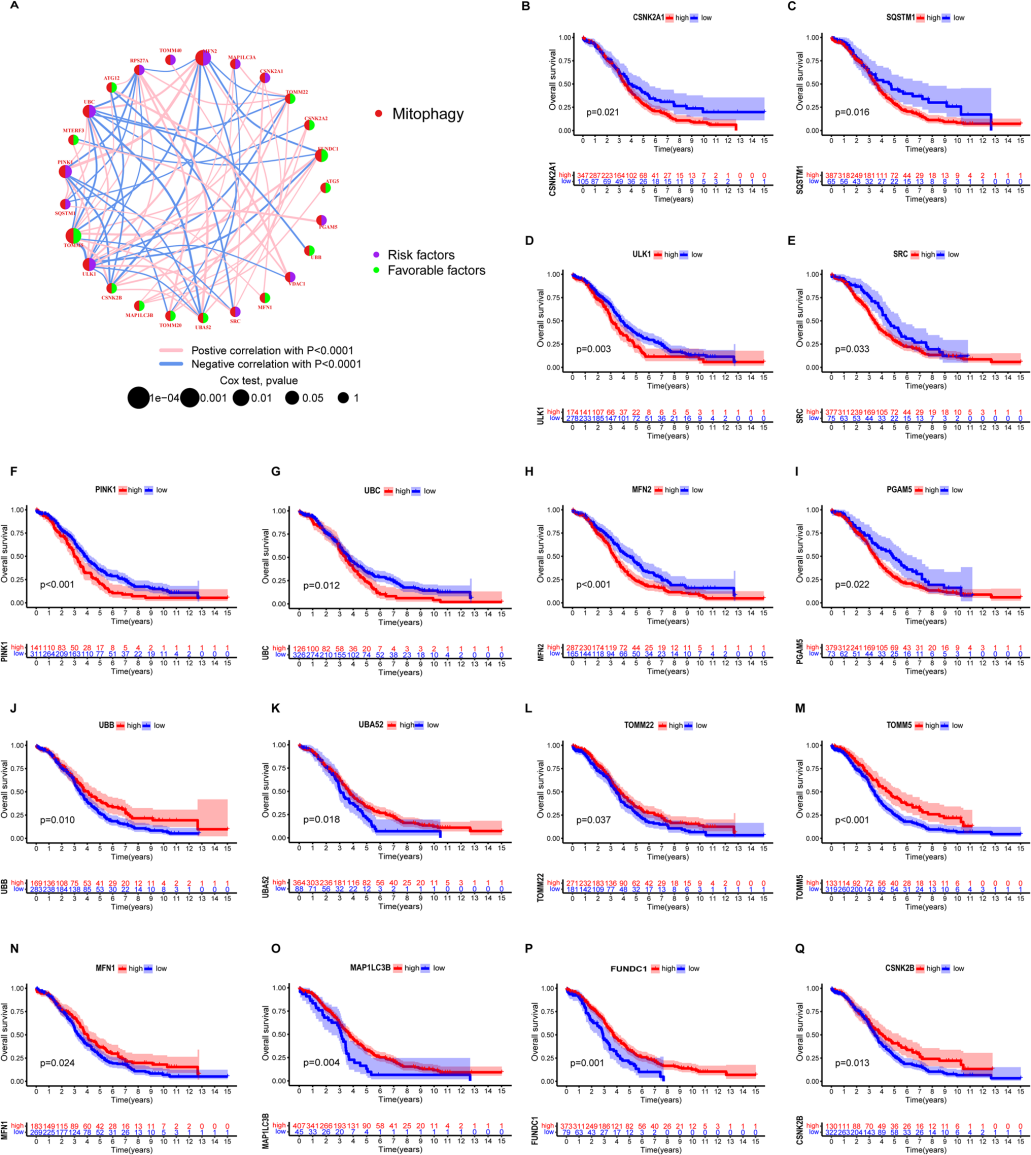
Correlation and prognostic analysis of MRGs in OV. **A** Correlation and prognostic value of MRGs in OV. The line connecting the MRGs represents their correlation, with the line thickness indicating the strength of the correlation between MRGs. Blue and pink represent negative and positive correlations, respectively. **B**–**Q** Kaplan–Meier analysis of indicated MRGs in OV

To comprehensively explore the expression profile of mitophagy-related genes (MRGs) in ovarian cancer (OV) development, we merged two datasets, TCGA-OV and GSE63885, comprising data from 480 patients with OV. We conducted correlation and univariate regression analyses to reveal the correlation of MRGs with OV development and their prognostic values (Fig. 4A). Our analysis showed that MFN2 and PINK1 were positively correlated and represented risk factors for OV development. Conversely, UBC and UBA52 were negatively correlated with each other, with UBC being a risk factor for OV, while UBA52 was protective against OV. Kaplan–Meier analysis indicated that high expression levels of CSNK2A1, SQSTM1, ULK1, SRC PINK1, UBC, MFN2, and PGAM5OV correlated with worse overall survival (OS) in OV patients (Figs. 4B–I). In contrast, high expression levels of UBB, UBA52, TOMM22, TOMM5, MFN1, MAP1LC3B, FUNDC1, and CSNK2B correlated with better OS in OV patients (Fig. 4J–Q).

**Fig. 5:**
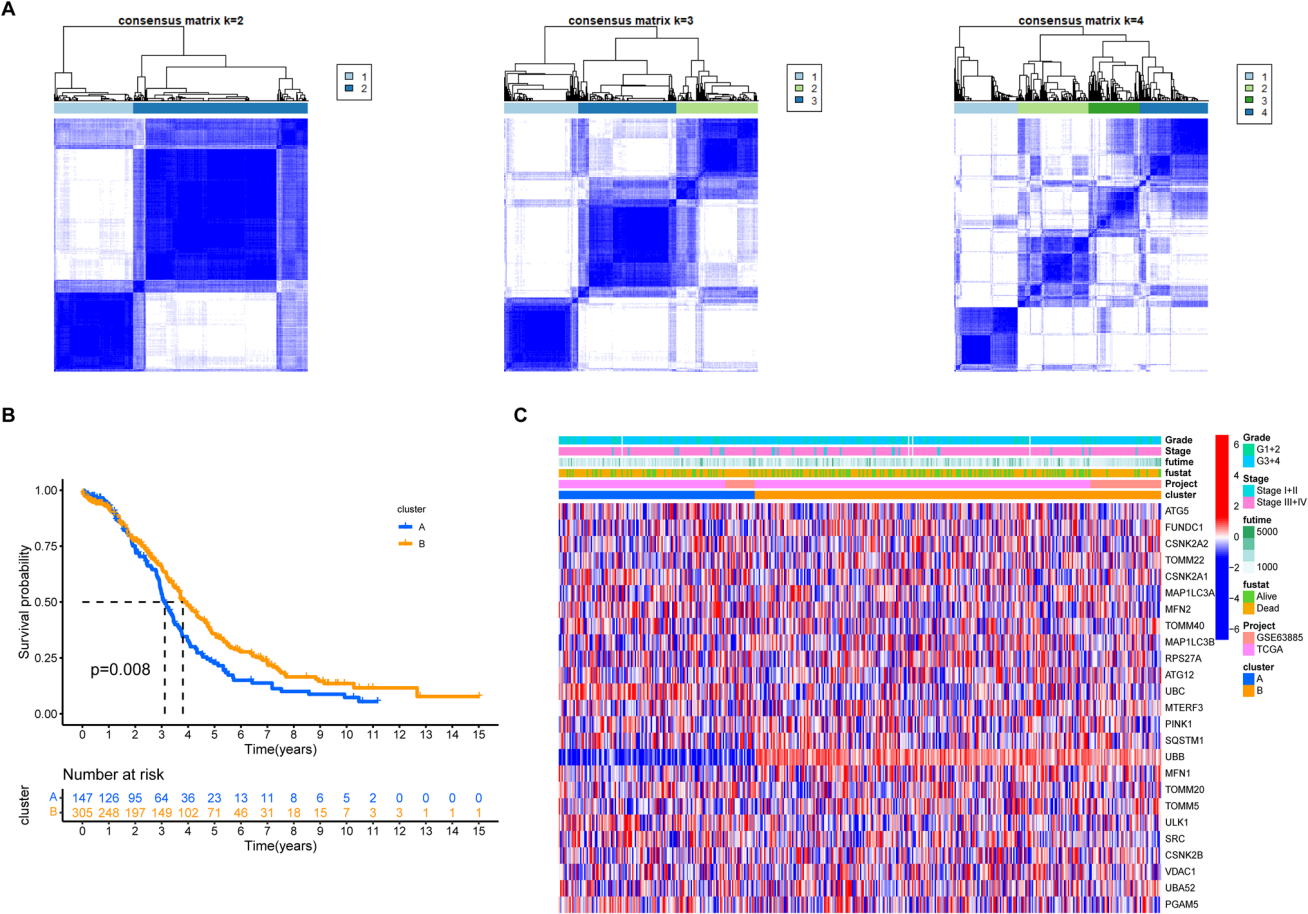
Identification of subtypes based on MRGs. **A** Consensus matrix heatmap defining various clusters (k = 2, 3, 4) and their correlation area. **B** Kaplan–Meier analysis of OV patients in two clusters. **C**. Distributions of clinical features and expression levels of MRGs between the two clusters

To further understand the expression characteristics of MRGs, we conducted the first unsupervised clustering of all patients based on the expression profiles of MRGs. Our analysis identified two subtypes of cluster A (n = 150) and cluster B (n = 330) as the most suitable classification state (Fig. 5A). There was a significant survival difference between the two clusters (Fig. 5B); patients with subtype A had a shorter OS than those with subtype B (log-rank test, p = 0.008). Figure 5C displays the distribution of clinical features and expression of MRGs in the two clusters.

**Fig. 6:**
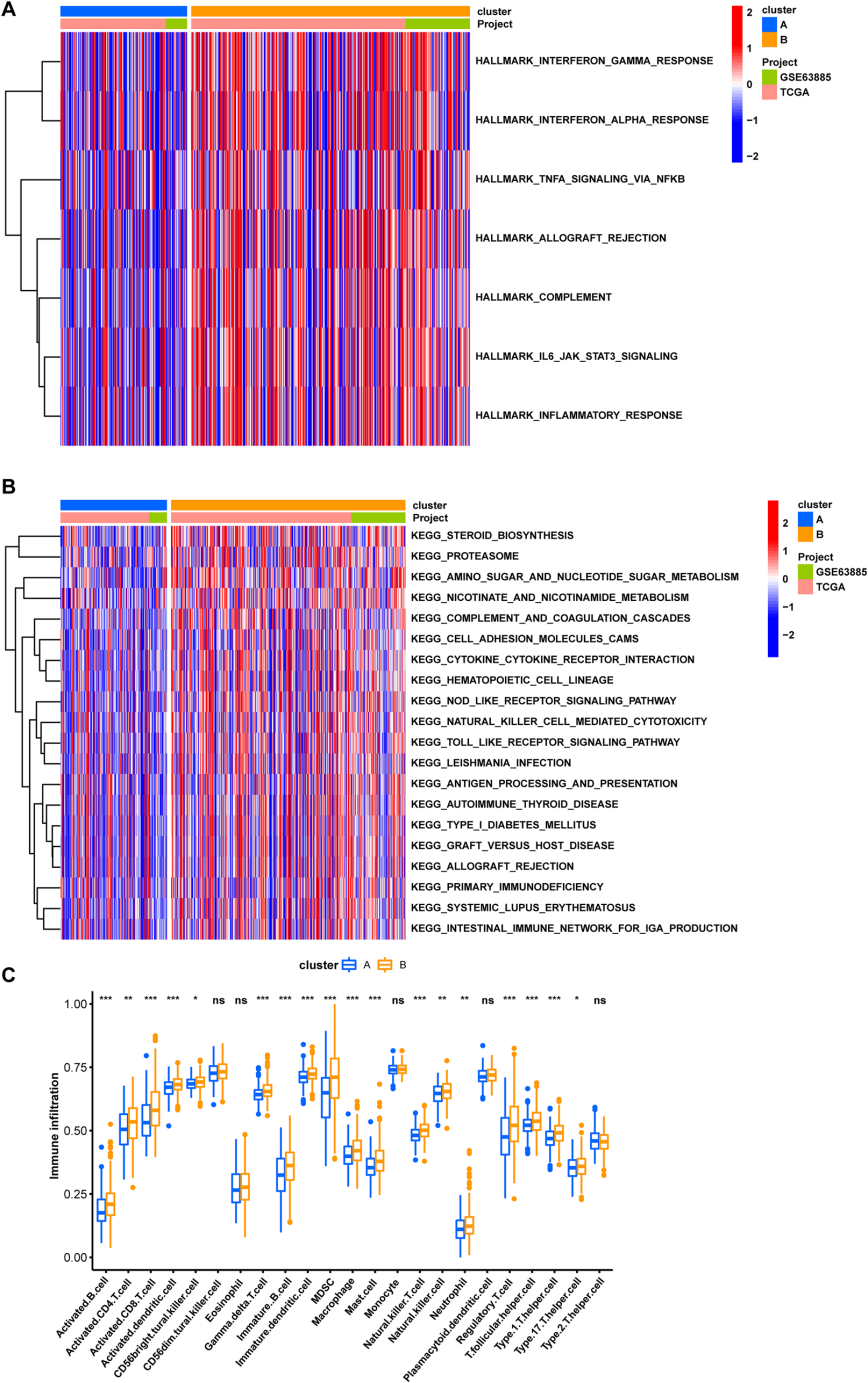
The difference between the two subtypes. **A** The score of HALLMARK pathways in two clusters. **B** The score of KEGG pathways in two clusters. **C** The difference of immune cell infiltration level in two clusters. *P < 0.05, **P < 0.01, ***P < 0.001, ****P < 0.0001

Additionally, we conducted Gene Set Variation Analysis (GSVA) to reveal the differences between the two clusters. We found that immune-related pathways, including interferon_gamma_response, interferon_alpha_response, TNFa_signaling_via_NFKB, and inflammatory_response pathways, had higher scores in cluster B, indicating a relatively immune-activated microenvironment (Fig. 6A). We observed similar results for immune-related pathways, including natural killer cell-mediated cytotoxicity, antigen processing and presentation, cytokine receptor interaction, primary immunodeficiency, intestinal immune network for IGA production, NOD-like, and toll-like receptor signaling pathways, which had higher scores in cluster B (Fig. 6B). Our analysis suggests that the greatest difference between the two subtypes lies in the altered immune microenvironment. To further evaluate the infiltration levels of immune cells in the two subtypes, we used the ssGSEA function of R-package GSVA to estimate the fraction of infiltrating immune cells (Fig. 6C). Our analysis revealed significant differences in the infiltration levels of most immune cells between the two subtypes, with 18 of the 23 immune cells highly infiltrating into the tumor in cluster B.

### Identification of gene subtypes based on representative DEGs

**Fig. 7:**
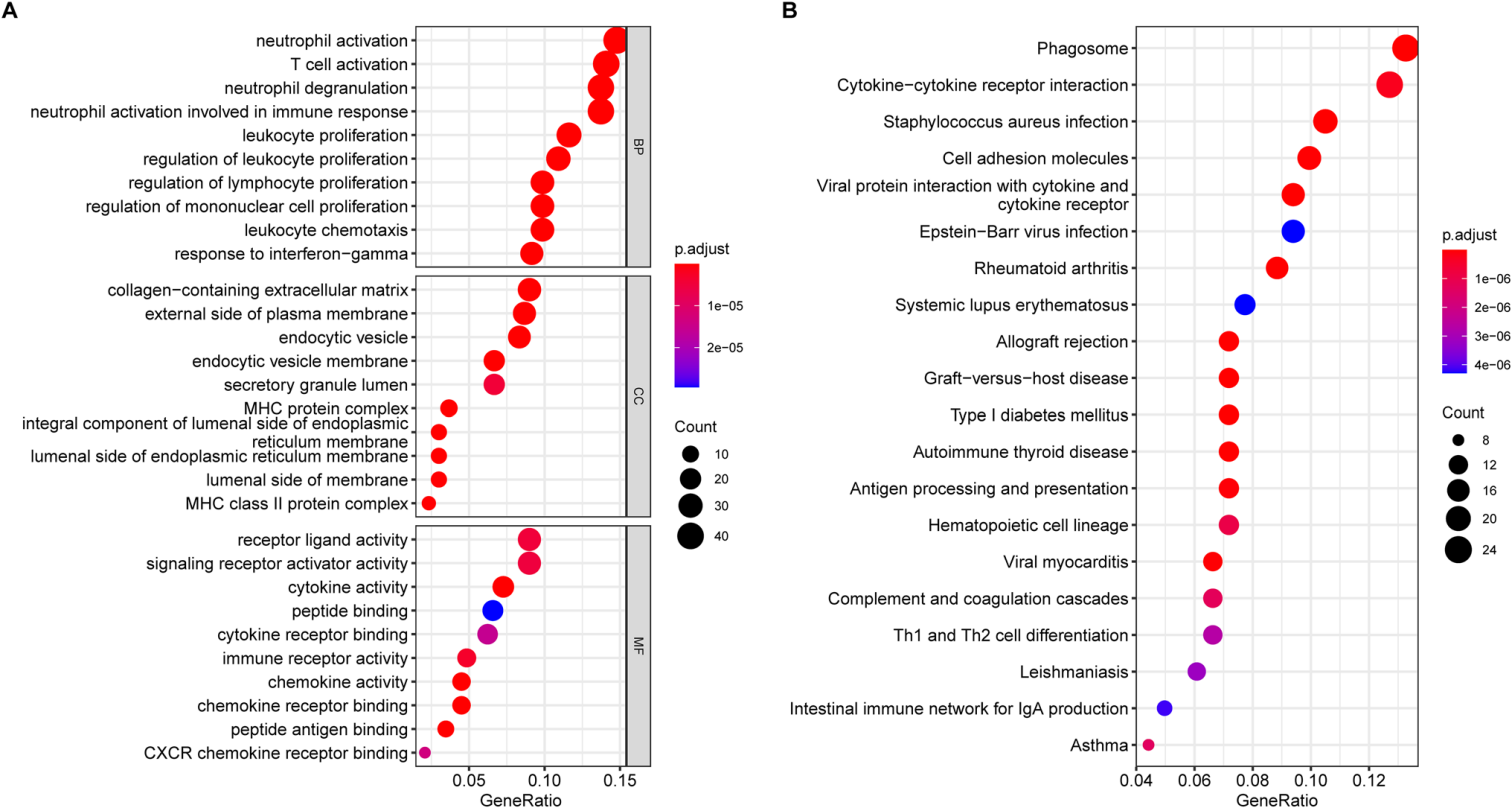
Enrichment analysis. **A** GO enrichment analysis of DEGs, including BP, CC, and MF. **B** Top20 terms of KEGG results

In order to investigate the biological behavior of different mitophagy patterns, we conducted a screening of 303 DEGs from two subtypes using the "limma" package [28] (Supplementary Table 3). The results of the GO enrichment analysis showed that the DEGs were significantly enriched in immune-related neutrophil and T cell activation in Biological Process, collagen-containing extracellular matrix in Cellular Component, and receptor ligand activity in Molecular Function (Fig. 7A). Meanwhile, the KEGG analysis revealed that the DEGs were enriched in phagosome and cytokine-cytokine receptor interaction pathways (Fig. 7B). These findings suggest that mitophagy plays a crucial role in regulating the tumor microenvironment (TME) and indicate that the DEGs are true mitophagy-related genes (MRGs).

**Fig. 8:**
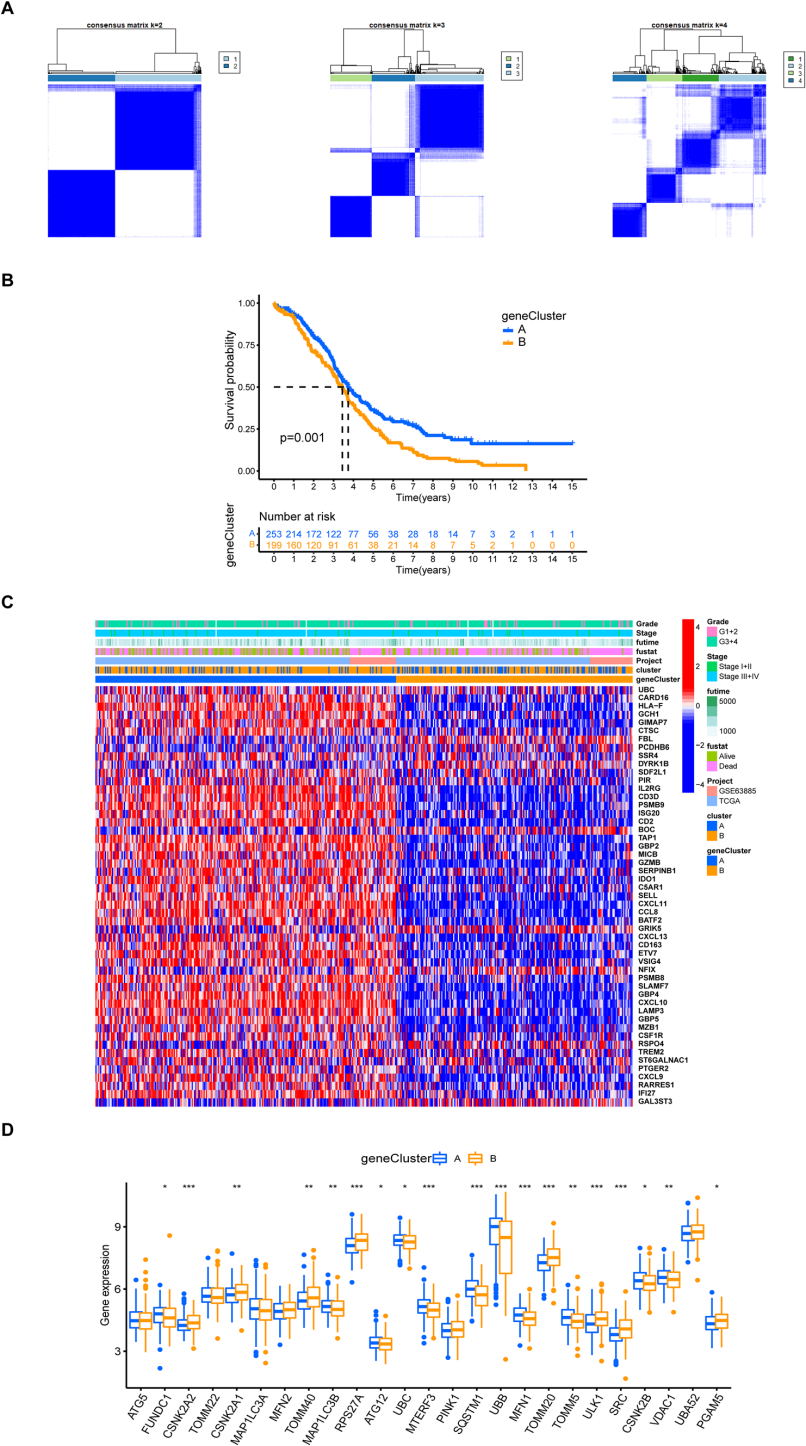
Identification of subtypes based on DEGs. **A** Consensus matrix heatmap defining various clusters (k = 2, 3, 4) and their correlation area. **B** Kaplan–Meier analysis of OV patients in two geneClusters. **C**. Distributions of clinical features and expression levels of 51 DEGs between the two geneClusters. **D** The expression of MRGs in two geneClusters. *P < 0.05, **P < 0.01, ***P < 0.001, ****P < 0.0001

To identify the prognostic value of the 303 DEGs, we performed univariate Cox regression analysis and identified 51 genes that were significantly related to overall survival (OS) (Supplementary Table 4). These 51 DEGs were considered as representative MRGs. To validate these results, we performed a second unsupervised clustering analysis based on the 51 prognostic genes, which divided patients into two subtypes, namely mitophagy geneCluster A and B (Fig. 8A). The results showed that patients with OV in geneCluster A had better OS than those in geneCluster B (Fig. 8B). The distribution of clinical features and expression of the 51 prognostic genes in the two geneClusters were also analyzed (Fig. 8C). GeneCluster A was associated with early-stage OV and longer OS. Moreover, we observed a distinct differential expression of most MRGs between the two geneClusters, which was consistent with the expected results of mitochondrial autophagy pattern (Fig. 8D). These findings suggest that mitophagy-related genes may serve as potential prognostic markers for ovarian cancer.

### Construction of the MRG_score

**Fig. 9:**
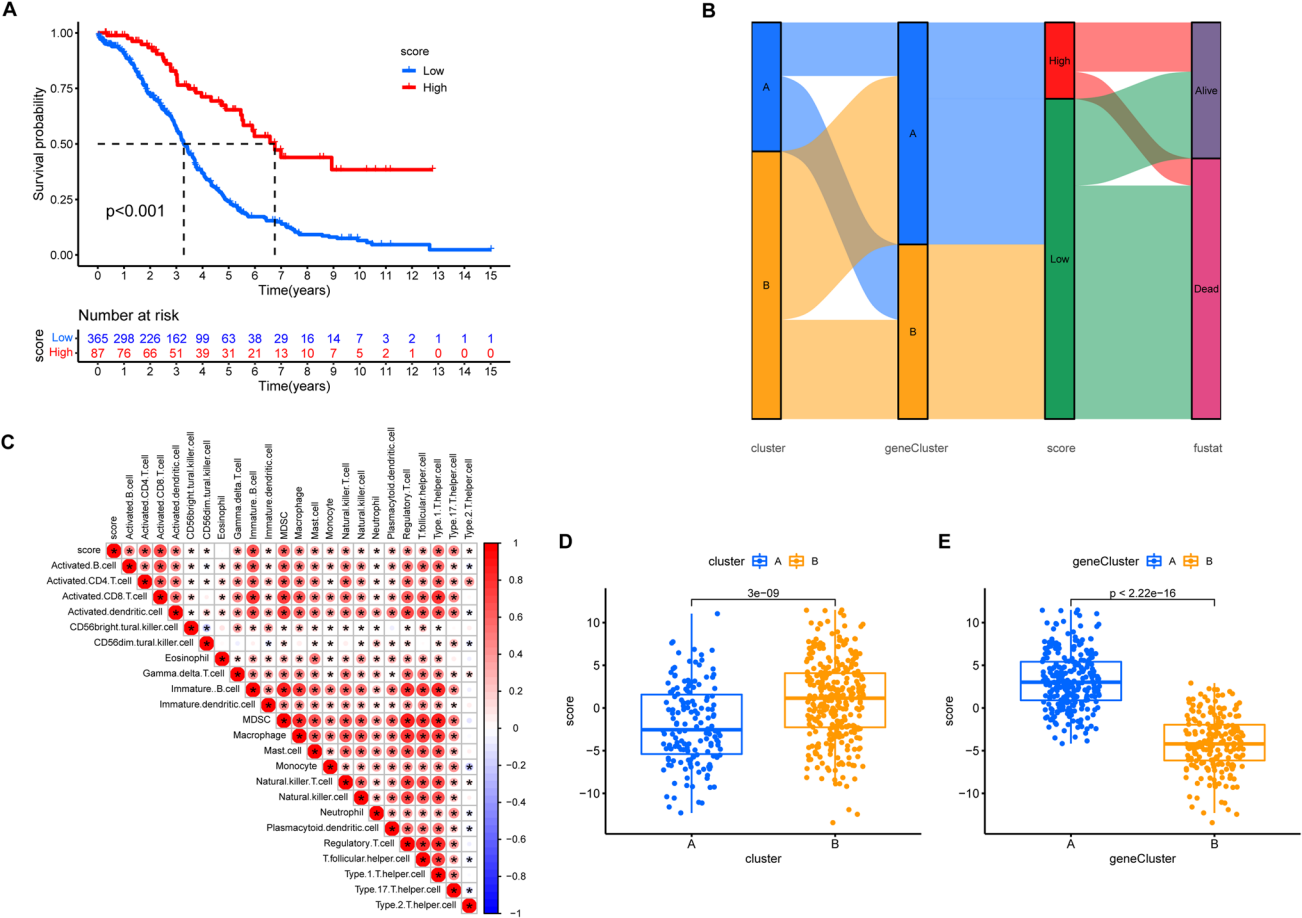
Construction of MRG_score. **A** Kaplan–Meier analysis of MRG_score in OV. **B** The sankey diagram visualized the correlation between cluster, geneCluster, MRG_score, and survival status of OV patients. **C** The correlation between MRG_score and immune cell infiltration. Red color represents positive correlation, Blue color represents negative correlation. **D** The MRG_score in cluster A and cluster B. **E** The MRG_score in geneCluster A and geneCluster B

Patients with immune-activated tumor microenvironment (TME) have shown improved survival and heightened sensitivity to immune checkpoint inhibitor (ICI) treatment [41, 42]. To evaluate the risk of patients with ovarian cancer (OV) and to identify those who might be receptive to ICI treatment, we developed the MRG_score based on 51 prognostic genes using a principal component analysis (PCA) algorithm. We found that patients with high MRG_scores have better overall survival (OS) (Fig. 9A). A Sankey diagram was used to illustrate the relationship between clusters, geneClusters, MRG_scores, and OS (Fig. 9B). Furthermore, we observed a positive correlation between the MRG_score and immune cell infiltration, particularly lymphocyte infiltration, indicating that the MRG_score may have predictive value for immunotherapy (Fig. 9C). In terms of cluster and geneCluster analyses, patients in cluster B or geneCluster A exhibited higher MRG_scores (Fig. 9D–E).

**Fig. 10:**
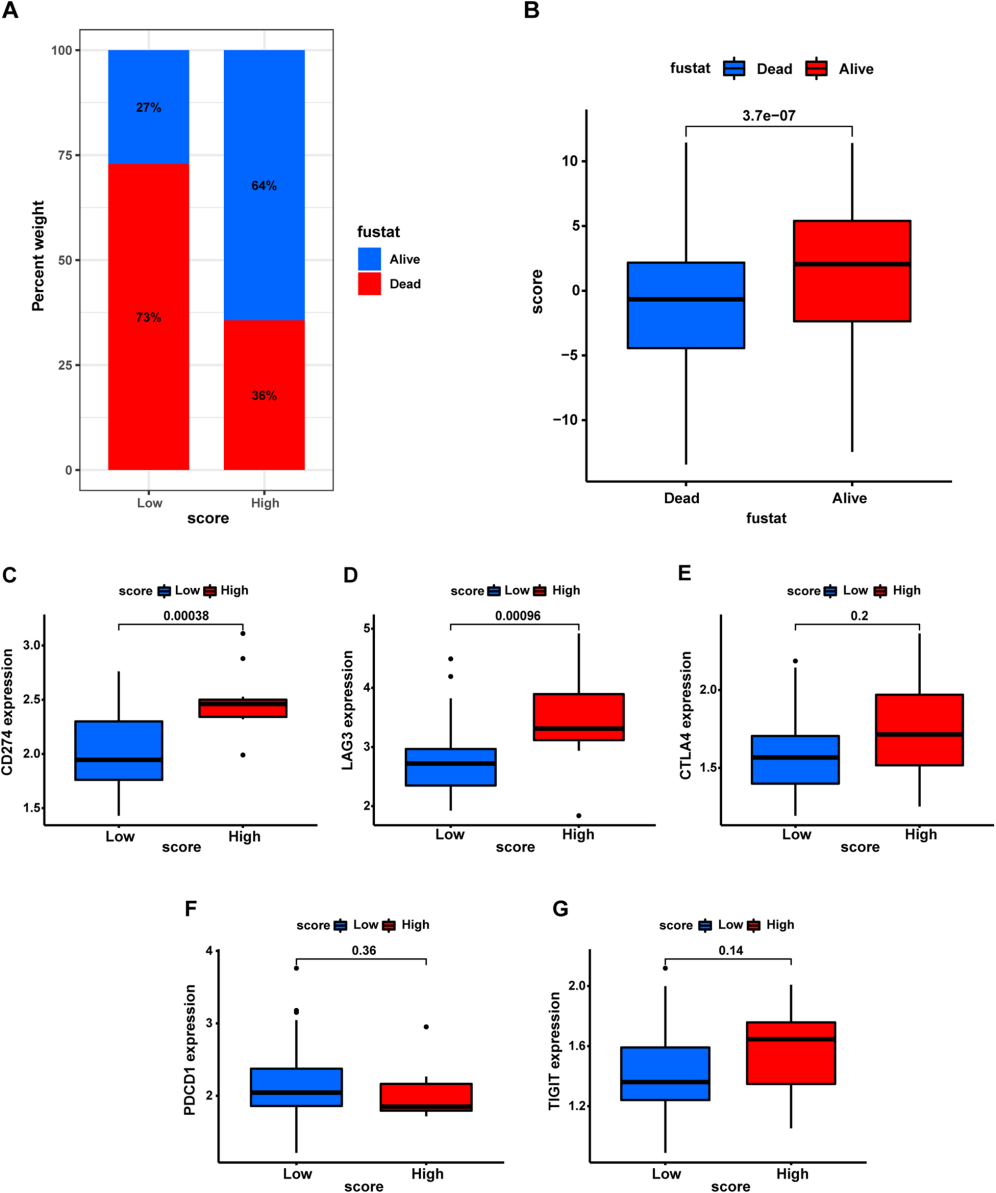
The association of MRG_score with survival status and immune checkpoint. **A** The percentage of alive and dead patients in high and low MRG_score groups. **B** The MRG_score in alive and dead groups. **C**–**G** The expression of indicated immune checkpoints in high and low MRG_score groups

Moreover, the proportion of surviving patients in the high MRG_score group (64%) was significantly higher than that in the low MRG_score group (27%) (Fig. 10A). Additionally, living patients had higher MRG_scores than deceased patients (Fig. 10B). These findings suggest that the MRG_score could serve as a useful tool for predicting ICI treatment sensitivity and improving clinical outcomes for patients with OV.

### The role of the MRG_score in predicting efficiency of ICI treatment

Tumor cells have the ability to evade recognition and elimination by the immune system through activation of immune checkpoint receptors, which is known as tumor immune escape. Therefore, the inhibition of immune checkpoint receptors has emerged as a novel approach in tumor immunotherapy. Several mature immune checkpoint receptors, such as programmed death protein 1 and its ligand (PD-1/PD-L1) and cytotoxic T lymphocyte-associated antigen-4 (CTLA-4), have been extensively studied [43–45]. Apart from PD-1/PDL1 and CTLA-4, numerous stimulatory and inhibitory co-receptors are involved in regulating T cell activation. Lymphocyte activation gene 3 (LAG3) is expected to become another significant target for chemotherapy following PD-1 [46–48]. Recent research indicates that the higher the expression of an immune checkpoint receptor, the better the efficacy of ICI treatment [49, 50]. In patients with high MRG_scores, immune checkpoint receptors, particularly CD274 (PD-L1) and LAG3, were found to be highly expressed (Fig. 10C–G), indicating that these patients may benefit significantly from anti-PD-L1 and LAG3 inhibitor therapy.

**Fig. 11:**
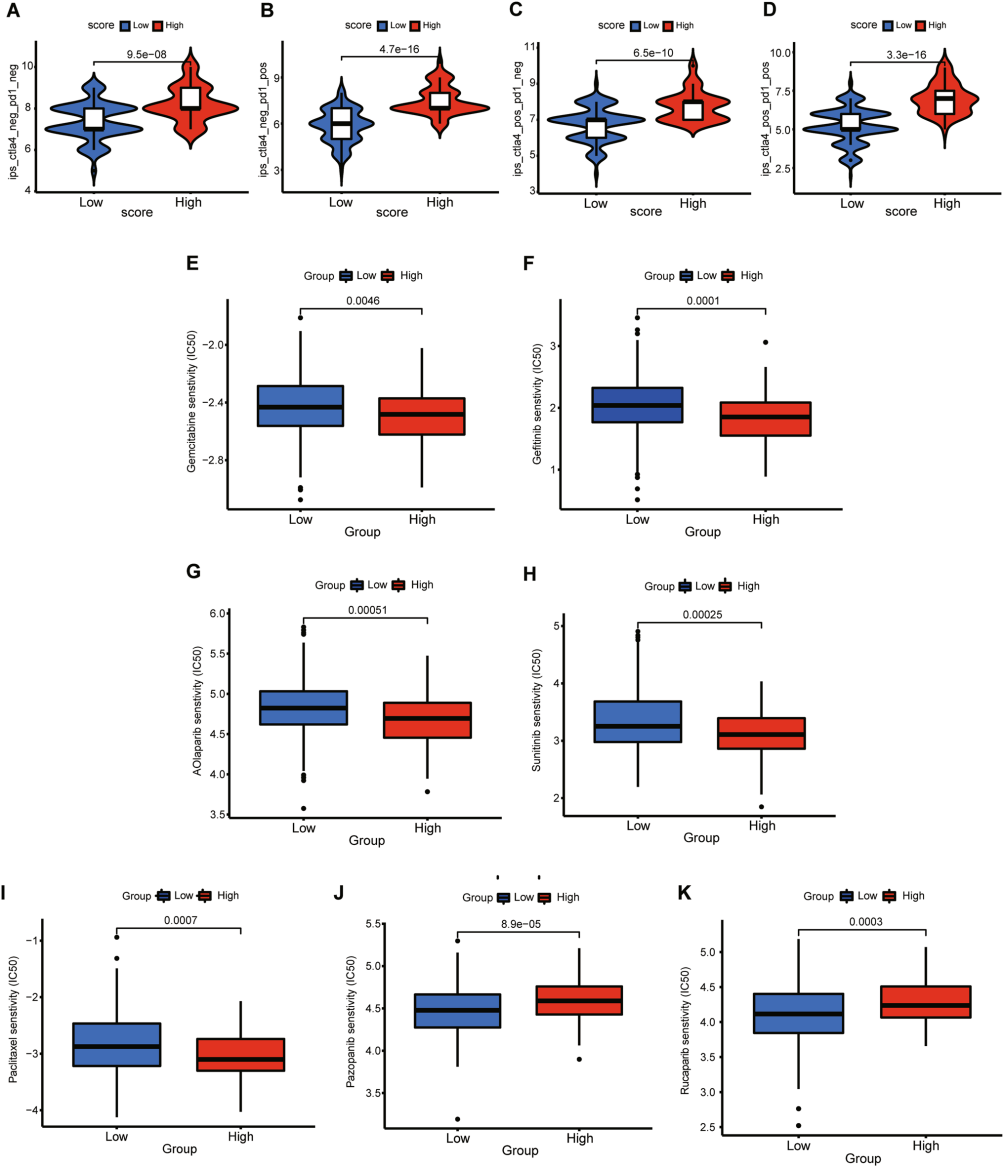
The Role of MRG_score in predicting efficiency of ICI treatment and drug sensitivity analysis. **A–D** The indicated IPS score of OV patients in high and low MRG_score groups. **E**–**K** The correlation of MRG_score with the sensitivity of chemotherapy agents (paclitaxel, gemcitabine) and targeted inhibitors (PARPi, tyrosine kinase inhibitor) commonly used for the treatment of OV

Since there is no publicly available immunotherapy dataset for OV, the immunophenoscore obtained from the TCIA database was used to predict the efficacy of ICI treatment. The findings reveal that patients with high MRG_scores are more responsive to ICI treatment (Fig. 11A–D). These results suggest that the MRG_score has the potential to predict the efficacy of ICI treatment for OV.

### MRG_score and drug sensitivity analysis

We also examined the correlation between the MRG_score and the effectiveness of chemotherapy agents (paclitaxel, gemcitabine) and targeted inhibitors (such as poly-ADP-ribose polymerase inhibitors (PARPi) and tyrosine kinase inhibitors) commonly utilized to treat ovarian cancer. Notably, patients with elevated MRG_scores displayed lower IC50 values for paclitaxel, gemcitabine, olaparib, gefitinib, and sunitinib. In contrast, individuals with low MRG_scores had significantly reduced IC50 values for pazopanib and rucaparib. These findings suggest that the MRG_score may be linked to drug sensitivity and could aid in identifying ovarian cancer patients who would benefit from chemotherapy and targeted drugs (as depicted in Fig. 11E–K).

## Discussion

The discovery of mitophagy has revealed its significant role in various biological processes, including immune cell development, activation, differentiation, inflammation, and tumor progression and metastasis. Recent research has suggested that mitophagy can aid tumor cells in evading immune surveillance. Studies have shown that changes in mitochondrial morphology can lead to reduced mitophagy, resulting in the apoptosis of immune cells [51]. However, other studies have demonstrated that mitophagy can promote immune escape through the Warburg effect, ultimately promoting tumor development [52]. Mitophagy also regulates the adaptive immune response by activating dendritic T cell synapses, CD8 + T cells, and memory natural killer cells, and it appropriately controls the immune response to prevent immune cells from clearing cancer cells [53]. Despite this, the relationship between mitophagy, immune cells, and cancer remains unclear. Furthermore, there is a lack of research on the effects of mitophagy on immunity in ovarian cancer, with the few available reports focusing only on single MRG-related genes or a single type of immune cell. Therefore, it is crucial to analyze multiple MRGs and evaluate their impact on the tumor microenvironment in ovarian cancer.

Figure 3 shows the top 10 mutated MRGs in ovarian cancer, which were UBC, MFN2, MFN1, SRC, CSNK2A1, VDAC1, ATG5, MTERF3, PINK1, and RPS27A. These mutated genes have been proven to play a regulating role in ovarian cancer based on evidence provided by previous literature, and several representative. The regulatory roles of these mutated MRGs in ovarian cancer were discussed by taking several representative genes as examples. The inducible expression of a UBC (Ubiquitin C)-targeting shRNA led to tumor regression and significant long-term survival benefits in orthotopic ovarian tumors, demonstrating the potential of targeting UBC in treating ovarian cancer [54]. MFN2 (Mitofusin 2) gene was found to promote mitophagy in ovarian cancer by increasing mitochondrial fusion and autophagosomal engulfment, and further leading to the activation of key autophagy-related proteins such as LC3B and p62 and results in the degradation of damaged mitochondria [55]. The mediating role of MFN2 in mitophagy reduces ROS levels and suppresses ovarian cancer progression through the AMPK/mTOR/ERK signaling pathway [55]. Mitofusin 1 (MFN1), a protein localized in the mitochondrial outer membrane, is known to regulate mitochondrial dynamics and play a vital role in ovarian cancer progression via modulation of mitochondrial dynamics, metabolism, and response to chemotherapy [56]. Under hypoxic conditions, ovarian cancer cells were found to undergo metabolic reprogramming and activate MFN1-mediated mitochondrial fission, ultimately leading to reduced production of reactive oxygen species (ROS) within the mitochondria and an increase in the survival of such cells [56]. Furthermore, intervention approaches aiming to inhibit or interrupt MFN1-mediated mitochondrial fission have recently been found to enhance the cytotoxic effects of cisplatin, largely through ROS elevation and activation of apoptotic pathways [56]. Taken an example of SRC (SRC Proto-Oncogene, Non-Receptor Tyrosine Kinase) gene, studies suggest that increased SRC expression results in hypoxic resistance to paclitaxel in ovarian cancer cells, and its inhibition using FV-429 can reverse this resistance [57–59]. Additionally, alterations in SRC gene expression have been linked to ovarian cancer manifestation, with its expression levels being associated with the occurrence and development of the disease [57–59]. The high expression levels of CSNK2A1 are associated with poor prognosis and increased tumorigenicity in ovarian cancer [60]. The delivery of bioactive siRNAs targeting CSNK2A1 using fusogenic peptides has been shown to induce apoptosis in ovarian cancer cells in vitro and in vivo, suggesting a potential link between CSNK2A1 and mitochondrial function [60]. VDAC1 (Voltage Dependent Anion Channel 1) was shown to play a critical role in promoting cisplatin resistance in ovarian cancer by regulating the HSP70/HK2/VDAC1 signaling pathway. The upregulation of VDAC1 mediated by the overexpression of PGC1α along with HSP70 was found to result in the increased glycolysis and reduced mitochondrial function in ovarian cancer cells; indicating the potential of VDAC1 as a therapeutic target in ovarian cancer [61].

The present study aimed to investigate copy number variations (CNV) and single nucleotide variations (SNV) of 29 mitochondrial-related genes (MRGs) in 480 patients with ovarian cancer (OV). Our results showed that MFN1, MAP1LC3A, TOMM20, CSNK2A1, and SRC had significantly increased CNV, while UBB, MAP1LC3B, CSNK2A2, and TOMM22 displayed a significant decrease in CNV. Furthermore, the SNV analysis revealed that UBC had the highest frequency of SNV in OV. We classified the patients into two mitophagy subtypes (cluster A and B) based on the expression of 29 MRGs. Patients in cluster A presented with worse clinical characteristics and poor overall survival (OS) compared to those in cluster B. To further understand the differences between the two MRG-related subtypes, we conducted gene set variation analysis (GSVA). The results in Fig. 6A, B shows that immune-related pathways (e.g., interferon_gamma_response, interferon_alpha_response, TNFα_signaling_via_NFKB, IL6_JAK_STAT3, natural killer cell-mediated cytotoxicity, antigen processing and presentation, cytokine receptor interaction, NOD-like receptor, and Toll-like receptor (TLR) signaling pathways) presented higher scores in cluster B while lower scores in cluster A. The regulatory role of natural killer cell-mediated cytotoxicity pathway in the tumor immunity of ovarian cancer has been highlighted in several previous literature [62–66]. NK cells are responsible for recognizing and killing malignant cells through direct cytotoxic activity or through the secretion of cytokines; thereby being regarded as a key determinant of tumor progression and response to therapy [67]. The combination therapy with cisplatin and NK cells has been shown to overcome immunoresistance in cisplatin-resistant ovarian cancer [66]. In addition, manipulating TLR signaling might hold great promise for the development of effective immunotherapeutic strategies in ovarian cancer. The activation of TLRs signaling pathway, particularly TLR2 and TLR4, by their ligands, such as bacterial products and damage-associated molecular patterns (DAMPs), was found to promotes ovarian cancer cell invasion through the induction of matrix metalloproteinases (MMPs) and the activation of the NF-κB signaling pathway [68]. The role of TLR signaling in the regulation of tumor immunity in ovarian cancer is complex and context-dependent [69–73]: on the one hand, TLR signaling in ovarian cancer has been shown to suppress the anti-tumor immune response and promote immune escape by promoting the differentiation and expansion of Tregs, inhibiting the activity of effector T cells, and restraining the muturation of dendritic cells (DCs). TLR signaling also induces the production of immunosuppressive cytokines (e.g., IL-10, TGF-β, and indoleamine 2,3-dioxygenase (IDO)) and thus further suppress the anti-tumor immune response. On the other hand, TLR signaling enhances the production of proinflammatory cytokines and chemokines which activate immune cells (NK cells and DCs) and stimulate their recruitment to the tumor microenvironment. The JAK/STAT3 pathway is often upregulated in ovarian cancer, leading to an immunosuppressive tumor microenvironment that can promote tumor cell growth and survival, inhibits apoptosis, and enhances angiogenesis and immune evasion [74, 75]. IL-6 was found to activate the JAK/STAT3 pathway and could promote tumor metastasis in ovarian cancer by enhancing the proliferation, migration, and invasion of tumor cells [76]. Targeted inhibition of JAK/STAT3 pathway can lead to reversal of immunosuppression and activation of immune cells to attack the tumor, resulting in decreased tumor growth and enhanced anti-tumor immune response [77, 78].

The MRG_score was developed to evaluate the risk of ovarian cancer in patients and identify those who might benefit from immunotherapy or other treatments. Initially, we identified 303 differentially expressed genes (DEGs) between two subtypes of ovarian cancer. Pathway analysis indicated that these DEGs were associated with immune system activation, including neutrophil activation and T cell activation, indicating their significance as MRG-related genes. We further narrowed down the DEGs to 51 genes that were associated with overall survival (OS) and classified patients into two mitophagy gene subtypes, geneCluster A and B, based on these genes. GeneCluster A was linked to early-stage cancer and longer OS. The expression of most MRGs varied significantly between geneClusters A and B, indicating their potential role in ovarian cancer progression. We then established the MRG_score and observed that patients with high MRG_scores had better OS and higher immune cell infiltration, suggesting that MRGs play a crucial role in the formation of the tumor microenvironment (TME) and can help identify patients who would benefit from ovarian cancer treatment.

The study revealed that patients with high MRG_scores exhibit elevated expression of immune checkpoints, particularly CD274 and LAG3, which renders them more responsive to ICI treatment. Additionally, patients with high MRG_scores demonstrate heightened sensitivity to various drugs, including veliparib, methotrexate, paclitaxel, gemcitabine, laparib, gefitinib, and sunitinib. Conversely, patients with low MRG_scores exhibit more positive IC50 values for cytarabine, rucaparib, lucaparib, and pazopanib. These findings suggest that MRG_score is closely associated with the effectiveness of immunotherapy, chemotherapy, and targeted inhibitor therapy, and may serve as a valuable tool for guiding clinical treatment decisions.

It is worthwhile to highlight the limitation of the current research, which encompass the retrospective nature of the study, potential heterogeneity of MRGs expression across different tumor subtypes and stages, and lack of experimental validation to support the biological functions of MRGs and the observed correlations. Firstly, one major limitation of this research is the retrospective nature of the study, which relied on public datasets from TCGA and GEO databases for gene expression and data analysis. The study lacks additional independent datasets to support the independence of MRG expression patterns, as well as confirm the accuracy of the MRG_score and its predictive ability for immunotherapy response and drug sensitivity. Secondly, although the model has been curated with 29 MRGs, it is necessary to include newly identified MRGs to enhance the accuracy of MRG scores. Thirdly, this study lacks a cohort of patients with OV treated with immunotherapy to independently verify the predictive ability of the MRG_score against PD-L1 or other ICI immune responses. In addition, the MRG_scores were determined using only retrospective datasets from public databases, which may have been influenced by inherent case selection bias. Therefore, a series of prospective cohort studies or even larger multicenter clinical studies are necessary to confirm and improve the accuracy of MRG scoring patterns. Furthermore, the current study did not analyze the potential heterogeneity of MRG expression across different tumor stages, which may affect the predictive value of the MRG_score. Moreoever, the study did not provide experimental verification to support the biological function of MRGs and the mechanisms driving the observed correlations with immune response and drug sensitivity. Despite these limitations, this study provides a valuable platform for future research to further investigate the role of MRGs in ovarian cancer and develop more effective personalized treatment strategies.

To sum up, this study represents the initial thorough examination of MRGs, which uncovered their significant regulatory mechanisms that impact the TME, clinical characteristics, and ultimate prognosis of OV patients. Additionally, we formulated the MRG_score and verified its dependability in predicting patient outcomes and the effectiveness and sensitivity of ICI treatment, chemotherapy, and targeted therapy. These results underscore the crucial clinical relevance of MRGs and present fresh insights into devising customized immunotherapy approaches for OV patients.

## Conclusion

In conclusion, this study revealed the presence of two distinct subtypes of the ovarian cancer patients based on their MRGs expression profiles. The cluster B subtype presents to be immune-hot, and thus had better prognostic outcomes and was less aggressive; while the cluster A subtype presents to be immune-cold, and thus had worse prognostic outcomes and thus more aggressive. The identification of these subtypes can enable the development of more targeted and personalized approaches to ovarian cancer treatment.

### Supplementary Information

Below is the link to the electronic supplementary material.

Supplementary file 1—The schematic diagram of the present study. (TIF 163 KB)

Supplementary file 2 (XLSX 96 KB)

## Data Availability

The datasets presented in this study can be found in online repositories. The names of the repository/repositories and Accession Number(s) can be found in the article.
