# Supplementary figures and images for: Mitophagy genes in ovarian cancer: a comprehensive analysis for improved immunotherapy
Source: Discov Oncol. 2023 Dec 1;14:221. doi: 10.1007/s12672-023-00750-y (PMC10692064; doi:10.1007/s12672-023-00750-y)

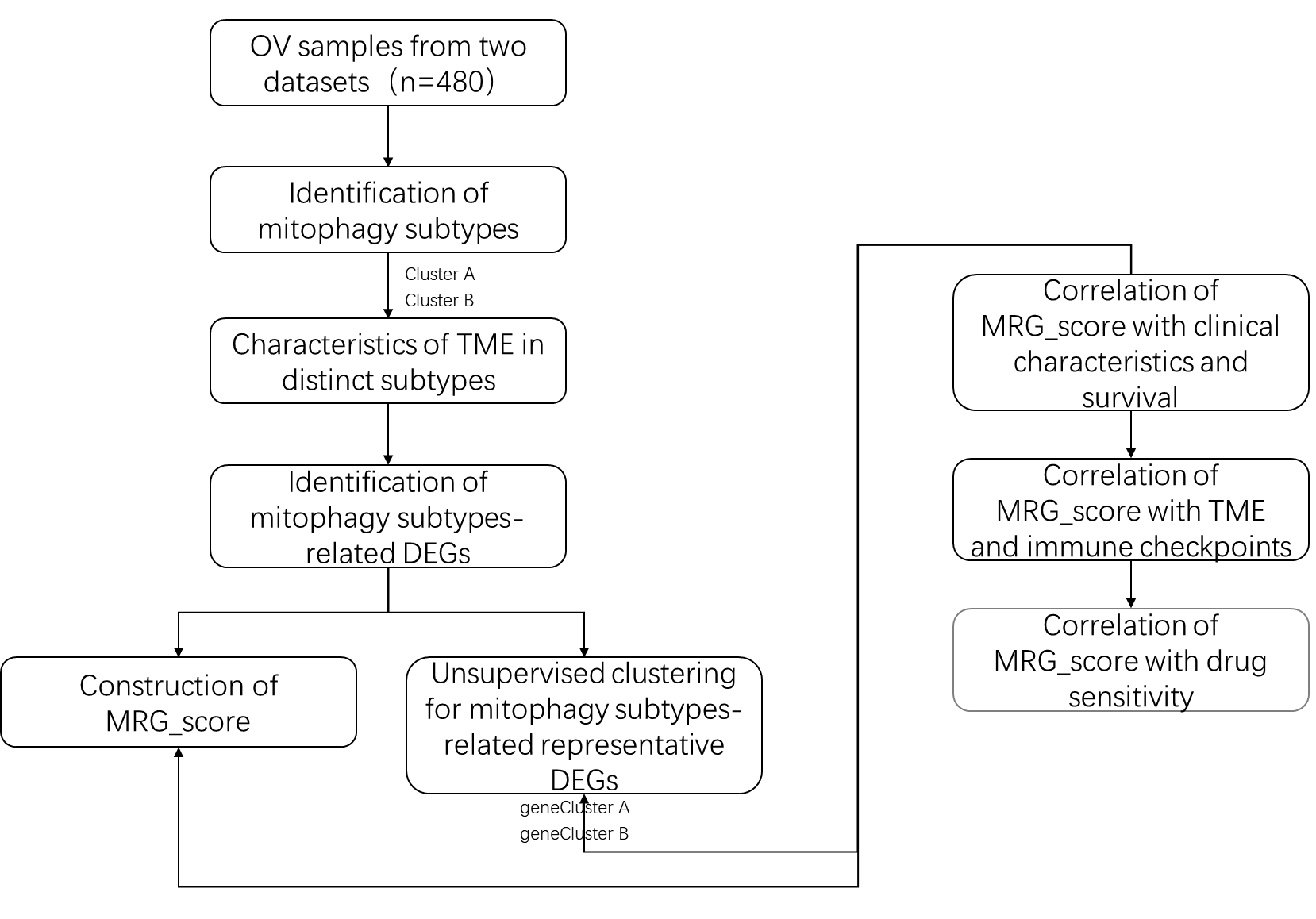

Supplement: Supplementary file 1 — The schematic diagram of the present study. (TIF 163 KB) [file 12672_2023_750_MOESM1_ESM.tif]
